# Preoperative Neutrophil-to-Lymphocyte Ratio as a Predictive Marker of Postoperative Infectious Complications in Pediatric Patients With Acute Appendicitis

**DOI:** 10.7759/cureus.71353

**Published:** 2024-10-13

**Authors:** Chandni, Abdul Wasay, Muhammad Amjad Chaudhary, Muhammad Umer Qureshi

**Affiliations:** 1 Pediatric Surgery, The Children's Hospital, Pakistan Institute of Medical Sciences, Islamabad, PAK; 2 General Surgery, Polyclinic Hospital, Islamabad, PAK

**Keywords:** appendicitis, complications, neutrophil-to-lymphocyte ratio (nlr), paediatric, platelet-to-lymphocyte ratio (plr)

## Abstract

Background

Several studies have investigated the role of neutrophil-to-lymphocyte ratio (NLR) in diagnosing and predicting the severity of acute appendicitis; however, few studies have analyzed its usefulness in pediatric appendicitis patients particularly in predicting postoperative complications. We investigated the role of NLR and assessed its clinical utility as a predictor of postoperative infectious complications in children with acute appendicitis.

Methodology

We performed a prospective cross-sectional study from July 2023 to July 2024 on 135 pediatric patients aged five to 12 years undergoing emergency appendectomy and having operative findings or histopathological confirmation of appendicitis. NLR was calculated from differential leucocyte count at the time of presentation to the emergency department. Patients were followed for one month after surgery for the development of complications. Univariate and multiple logistic regression was used to identify clinical factors significantly associated with postoperative complications. The sensitivity and specificity of these parameters to predict complications were determined using receiver operating characteristic curves.

Results

The average age was 8.36 ± 2.38 years. There were 36 (26.67%) laparoscopic and 99 (73.33%) open appendectomies. Eighty-three (61.48%) patients had simple while 52 (38.52%) had complicated appendicitis. NLR, platelet-to-lymphocyte ratio (PLR), and C-reactive protein (CRP) were significantly higher in patients with complicated appendicitis (p < 0.05). Twenty-nine patients (21.48%) developed complications like wound infection (n = 29, 21.48%), wound dehiscence (n = 5, 3.7%), prolonged ileus (n = 12, 8.89%), intra-abdominal abscess (n = 7, 5.19%), and early adhesive obstruction (n = 4, 2.96%). Univariate and multiple logistic regression analysis of the clinical parameters revealed that NLR (OR: 1.28; 95% CI: 1.004-1.64), PLR (OR: 1.01, 95% CI: 1.001-1.01), and type of appendicitis (OR: 7.11; 95% CI: 1.57-32.22) had an independent significant association with all postoperative complications (p < 0.05). NLR and PLR presented an area under the curve (AUC) of 0.86 (p < 0.001) and 0.83 (p < 0.001), respectively, which was significantly higher than the AUC of neutrophil count (AUC: 0.66, p = 0.006), lymphocyte count (AUC: 0.80, p < 0.001), CRP (AUC: 0.70, p = 0.001), and plasma sodium levels (AUC: 0.64, p = 0.006). NLR and PLR showed the highest sensitivity (79.3% and 79.3%) and specificity (95% and 82.1%) at cut-off values of 9.53 and 166.01, respectively.

Conclusion

The NLR can be used for distinguishing complicated from uncomplicated appendicitis, for prioritizing cases for operative management, and for identifying patients likely to experience complications after surgery.

## Introduction

Acute abdomen secondary to appendicitis remains the most common surgical emergency in children comprising 1-2% of all pediatric surgical admissions overall. Early diagnosis remains challenging in children due to difficulties in doctor-patient communication and a lack of classical symptoms that overlap with many other common childhood illnesses [1]. The spectrum of disease severity ranges from simple catarrhal or phlegmonous appendix to complicated appendicitis comprising of gangrenous, perforated, or autoamputated appendix with localized or diffuse abscess or an appendicular mass. Misdiagnosis leads to the progression of disease, which increases morbidity; on the other hand, having a low threshold for diagnosis is also not feasible as according to Jukić and colleagues, the negative appendectomy rate in children may be as high as 50% [2]. Appendectomy is a common surgical procedure with low mortality but carries a postoperative complication rate of 5-28%. There is an increased risk of postoperative complications in patients undergoing appendectomy for complicated appendicitis; therefore, it becomes crucial to appropriately diagnose this group. This does not mean that appendectomy for simple appendicitis does not carry a risk of potential postoperative complications, although they are likely to be lower in incidence [3]. Hughes et al. followed 266 patients after appendectomy and reported that postoperative intra-abdominal infections complicated 4.2% of simple appendicitis and 12.8% of complicated appendicitis hence leading to a significant increase in morbidity, longer hospital stays, and readmissions in some cases [4].

The role of clinical scoring and various laboratory parameters in diagnosing and distinguishing between simple and complicated appendicitis and predicting postoperative complications has been studied in the past years with varying results. The neutrophil-to-lymphocyte ratio (NLR) has been suggested as a sensitive marker of inflammation in inflammatory bowel disease, and in the prognosis of colorectal and gastric cancers [5-7]. Kahramanca and colleagues studied its role as a marker of acute appendicitis in adult patients suggesting that it may prevent negative appendectomy based on its predictive value [8]. To the best of our knowledge, there are few studies examining the value of NLR in treating children with appendicitis, particularly in predicting postoperative complications [8,9]. In our institution, which is a heavily burdened pediatric tertiary care center in a low-middle-income country rendering services to the city as well as the periphery, it becomes ever-crucial to identify patients at risk of following a complicated postoperative course. This strategy may help to optimize and prioritize patient care by timely discharge of the low-risk patients and continued surveillance of the higher-risk group to reduce morbidity and the burden and costs associated with readmission. The aim of this study was to analyze the role of preoperative NLR in predicting the development of postoperative complications particularly infectious after appendectomy in children.

## Materials and methods

Study design

A prospective cross-sectional study was performed on 135 patients who visited the Department of Pediatric Surgery, The Children's Hospital for acute appendicitis requiring surgery between July 2023 and July 2024 after obtaining approval from the Ethical Review Board (Approval Number: F.1-1/2015/ERB/SZABMU/1141) of our institution, the Pakistan Institute of Medical Sciences, Shaheed Zulfiqar Ali Bhutto Medical University. The WHO sample size calculator for diagnostic test accuracy study was used to calculate the sample size with a level of precision of 10% and a confidence interval (CI) of 95%.

Inclusion and Exclusion Criteria

All patients between the ages of five and 12 years undergoing emergency appendectomy and having operative findings suggestive of appendicitis or later confirmation by histopathological review were included. Due to physiological variations in hematological parameters and a different spectrum of clinical presentation in that age group, patients younger than five years were excluded from the study. Patients who did not have histological evidence of appendicitis or who underwent an interval appendectomy following antibiotic therapy were also excluded.

Demographic and clinical parameters recorded preoperatively included age, gender, weight, duration of symptoms, temperature at the time of presentation, ultrasound findings, and laboratory variables. Time to surgery, duration of surgery, type of procedure (open vs. laparoscopic) as well as operative findings were also recorded. A routine complete blood picture along with quantitative C-reactive protein (CRP) levels and serum electrolytes were performed for each patient at the time of presentation in the emergency department. The NLR was calculated by dividing the absolute neutrophil count by the absolute lymphocyte count. Preoperatively, catarrhal or phlegmonous appendix was defined as simple while gangrenous, perforated, autoamputated, or those with an associated peri-appendiceal abscess were classified as complicated appendicitis. Preoperative intravenous antibiotic therapy with ceftriaxone and metronidazole was started in all patients and continued 24 to 72 hours postoperatively with the addition of amikacin depending on operative findings. Patients with simple appendicitis were discharged the next day while those with complicated appendicitis remained in-patient for a minimum of 72 hours. Oral antibiotics were commenced for five to seven days at discharge in all cases. Postoperatively, patients were followed for a period of one month for surveillance of the development of complications such as superficial and/or deep surgical site infection, intra-abdominal abscess, wound dehiscence, cholangitis, enteritis, paralytic ileus, or early adhesive obstruction. Patients who did not develop any postoperative complication were classified as group A, while those encountering at least one complication postoperatively were classified as group B. Surgery was performed by a group of surgeons having comparable experience using the same instruments. Prior to surgery, the parent or guardian's informed written consent was obtained.

Statistical analysis

Results were analyzed using SPSS version 26 (IBM Corp., Armonk, NY). For quantitative data, mean and standard deviations were calculated while categorical data were expressed as frequency and percentages. The normality of the data was analyzed using the Kolmogorov-Smirnov test. For comparison between two groups, the Student’s t-test was used for normally distributed numerical data while the Mann-Whitney U test was used for skewed data. Categorical variables were compared using chi-square and Fisher’s exact tests. Univariate analysis using binary logistic regression was performed to identify variables strongly associated with the subsequent development of postoperative complications. The statistical significance of the variables identified by univariate analysis was further tested by multiple logistic regression. Odds ratios (OR) were calculated with 95% CIs. The relationships between systemic inflammatory variables and the development of postoperative complications were assessed by the area under the curves (AUCs) of the receiver operating characteristic (ROC) curves. The cutoff values for these variables were set closest to the (0,1) point on the ROC curves, corresponding to the maximum sensitivity and specificity for the prediction of complications. The level of significance was set at 0.05.

## Results

A total of 135 patients were included in the study, out of which 29 patients classified as group B developed one or more postoperative complications within the first month. The age range was five to 12 years (mean: 8.36 ± 2.38 years). Overall male-to-female ratio was 2.29. There were no negative appendectomies. Regarding clinical and hematological parameters, it was observed that group B patients had higher values of neutrophil count (p < 0.009), lymphocyte count (p < 0.001), and CRP (p < 0.001) on initial laboratory investigations. NLR and platelet-to-lymphocyte ratio (PLR) were also significantly higher in this group (p < 0.001). In terms of electrolytes, plasma sodium levels were significantly lower in group B patients (p = 0.01). No significant relationship between postoperative complications and the type of appendectomy was observed. There was a significant difference in operative findings with a higher incidence of complicated appendicitis in group B (p < 0.001). The clinical characteristics of both groups are summarized in Table 1.

**Table 1:** Comparison of demographic, clinical, and laboratory parameters of patients in groups A and B. Group A: Appendicitis with no postoperative complication. Group B: Appendicitis with at least one postoperative complication. Continuous variables are expressed as mean ± standard deviation. Categorical variables are expressed as frequency (n) and percentages. The level of significance was set at 0.05. * P-value obtained by comparing continuous data through Student's t-test and Mann-Whitney U test and categorical data by chi-square test and Fisher’s exact test.

Parameter	Group A (n = 106, 78.52%)	Group B (n = 29, 21.48%)	Test value	P-value*
Mean age (years)	8.21 ± 2.39	8.93 ± 2.31	1771.5	0.20
Gender (male/female)	73 (68.7%)/33 (31.13%)	21 (72.4%)/8 (27.5%)	0.13	0.71
Weight (kg)	30.28 ± 10.56	33.83 ± 10.07	1852.5	0.09
Duration of symptoms (hours)	3.02 ± 1.77	3.17 ± 1.56	1670.5	0.46
Temperature (°C)	37.86 ± 0.74	38.05 ± 0.83	1745.5	0.26
Hemoglobin (g/dL)	11.22 ± 1.76	11.23 ± 1.69	1539.5	0.98
Total leucocyte count (10^9^/L)	14.76 ± 5.28	16.14 ± 4.39	-1.43	0.15
Neutrophil count (10^9^/L)	11.26 ± 5.13	13.96 ± 3.81	-2.64	0.009
Lymphocyte count (10^9^/L)	2.9 ± 1.19	1.57 ± 1.37	4.74	<0.001
Platelet count (10^9^/L)	282.08 ± 100.37	296.69 ± 123.22	1665	0.49
Neutrophil-to-lymphocyte ratio (NLR)	4.87 ± 3.69	13.92 ± 8.83	2651.5	<0.001
Platelet-to-lymphocyte ratio (PLR)	119.48 ± 80.41	288.35 ± 170.26	2571	<0.001
C-reactive protein (CRP)	32.83 ± 20.22	49.93 ± 24.78	2166	0.001
Plasma sodium levels (mEq/L)	136.27 ± 4.35	133.93 ± 5.04	1088.5	0.01
Preoperative ultrasound findings (equivocal/positive)	69 (65.9%)/37 (34.9%)	18 (62.1%)/111 (37.93%)	0.09	0.76
Average time to surgery (hours)	3.43 ± 1.10	3.62 ± 1.29	1654	0.51
Average duration of surgery (minutes)	38.25 ± 11.85	42.59 ± 16.12	1714.5	0.32
Type of appendectomy (open/laparoscopic)	78 (73.58%)/28 (26.41%)	21 (72.41%)/8 (27.58%)	0.16	0.89
Operative findings (simple/complicated)	76 (71.69%)/30 (28.3%)	7 (24.13%)/22 (75.86%)	21.74	<0.001

Hematological parameters such as total leucocyte count (TLC), neutrophil count, NLR, PLR, and CRP were significantly higher in patients with complicated appendicitis on operative findings, whereas these patients also had lower preoperative hemoglobin and plasma sodium (Na) levels (p < 0.05).

Postoperative complications developed by the patients

**Figure 1:**
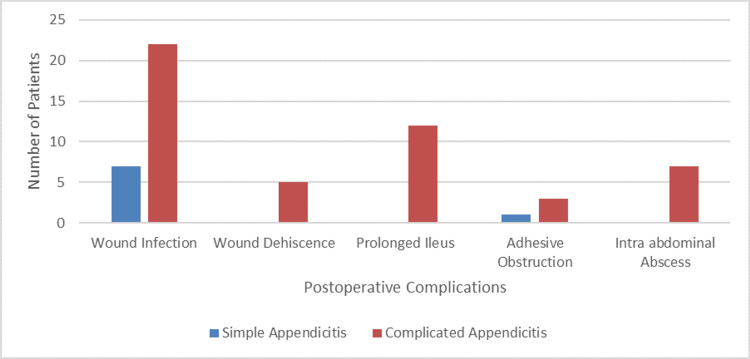
All postoperative complications developed by the patients.

Twenty-nine patients developed postoperative complications. Superficial surgical site infection was noted in all of them. There were nine readmissions. Seven patients revisited with acute abdominal pain and bowel obstruction within the first 10 days of surgery and were found to have an intra-abdominal collection on ultrasound. Two patients had small collections and were managed conservatively with intravenous antibiotics. Five patients were re-explored and found to have multiple pockets of pus with enteritis; these patients also had partial wound dehiscence at the time of presentation. One patient developed early small bowel obstruction after open appendectomy for the catarrhal appendix and failed conservative management; re-exploration revealed adhesions with appendiceal stump and wound site. Prolonged paralytic ileus was noted in 12 patients, and all had complicated appendicitis. There was no mortality. Complications developed by the patients are summarized in Figure 1.

Predictive factors for infectious complications in patients with acute appendicitis

Univariate binary logistic regression analysis of the various parameters revealed that the variables significantly associated with all postoperative complications were preoperative absolute neutrophil count (OR: 1.11; 95% CI: 1.02-1.21), lymphocyte count (OR: 0.36; 95% CI: 0.22-0.57), NLR (OR: 1.33; 95% CI: 1.19-1.49), PLR (OR: 1.01; 95% CI: 1.007-1.01), CRP (OR: 1.034; 95% CI: 1.01-1.05), plasma Na levels (OR: 0.896; 95% CI: 0.81-0.98), and type of appendicitis (OR: 7.96, 95% CI: 3.08-20.58) on operation (p < 0.05). Multiple logistic regression analysis of the identified significant factors identified NLR (OR: 1.28; 95% CI: 1.004-1.64), PLR (OR: 1.01, 95% CI: 1.001-1.01), and type of appendicitis (OR: 7.11; 95% CI: 1.57-32.22) as independent factors associated with all postoperative complications (p < 0.05).

Sensitivity and specificity analysis using ROC curves

While utilizing ROC curves for sensitivity and specificity analysis for the prediction of postoperative complications, we observed that NLR had an area under the curve (AUC) of 0.86 (p < 0.001), while PLR showed an AUC of 0.83 (p < 0.001), which were significantly higher than the AUC of the neutrophil count (AUC: 0.66, p = 0.006), lymphocyte count (AUC: 0.80, p < 0.001), CRP (AUC: 0.70, p = 0.001), and plasma Na levels (AUC: 0.64, p = 0.006). NLR and PLR showed the highest sensitivity (79.3% and 79.3%) and specificity (95% and 82.1%) at cut-off values of 9.53 and 166.01, respectively, while CRP levels had a low sensitivity of 62.1% and specificity of 64.2% at cut-off value of 37.5. Table 2 displays the parameters analyzed for the prediction of complications, their AUC, cut-off values, and the sensitivity and specificity associated with them.

**Table 2:** AUC and cut-off values (obtained by Youden Index) of the parameters analyzed for the prediction of postoperative complications. AUC: area under the curve; CI: confidence interval; NLR: neutrophil-to-lymphocyte ratio; PLR: platelet-to-lymphocyte ratio; CRP: C-reactive protein; Na: sodium.

Predictors	Cut-off value	Sensitivity	Specificity	AUC	95% CI	P-value
NLR	9.53	79.3%	95%	0.86	0.77-0.95	<0.001
PLR	166.01	79.3%	82.1%	0.83	0.74-0.93	<0.001
Neutrophil count (10^9^/L)	12.3	65.5%	60.4%	0.66	0.57-0.76	0.006
Lymphocyte count (10^9^/L)	2.13	75.9%	29.2%	0.80	0.69-0.92	<0.001
CRP	37.5	62.1%	64.2%	0.70	0.60-0.81	0.001
Plasma Na levels (mEq/L)	136.5	65.5%	46.2%	0.64	0.53-0.77	0.006

**Figure 2:**
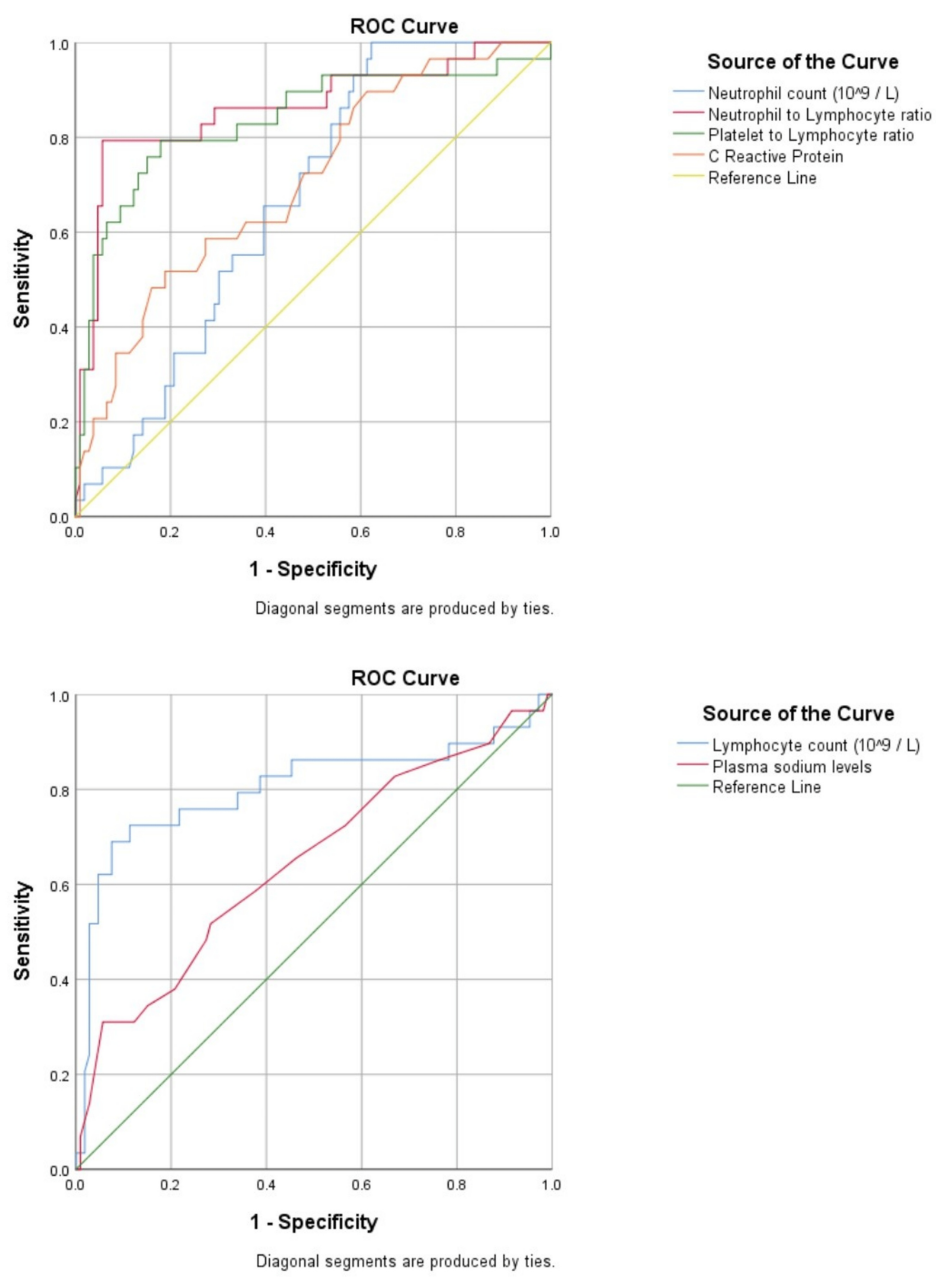
Optimal cut-off values of inflammatory variables determined by the receiver operating characteristic curves for distinguishing between group A and group B.

Figure 2 shows the ROC curves for the prediction of complications.

## Discussion

Although acute appendicitis is the most common cause of pediatric surgical admissions, diagnosis can be difficult at times despite advances in imaging, due to the high frequency of unusual presentations and symptoms. In the era of COVID-19, many institutions in our country have leaned toward nonoperative management (NOM) of acute uncomplicated appendicitis in children utilizing antibiotics. National studies have mostly favored this approach [10]. Since less than 19% of children develop complicated appendicitis, most patients with simple acute appendicitis can be treated with either nonoperative or operative measures [11]. In 2017, a systematic literature review comparing appendectomy to NOM for pediatric simple appendicitis showed that with an initial success rate ranging from 58% to 100% and a 0.1-31.8% recurrence at one year, NOM may be a viable choice in children given its cheaper costs, reduced morbidity, and fewer disability days compared to surgery [12]. However, in the presence of appendicolith, the failure rate of NOM becomes high, hence surgery is preferred in those cases [13-17]. Patient selection therefore becomes crucial as misdiagnosing complicated appendicitis while opting for NOM could result in potential morbidity and complications.

Studies in the literature have reported that the NLR may help in diagnosing acute appendicitis and in distinguishing complicated from simple appendicitis, which becomes useful when a management approach is to be decided [18]. Ishizuka et al. investigated the association between NLR and the prevalence of gangrenous and perforated appendicitis in 314 adult patients, establishing an NLR cut-off point >8, with a corresponding sensitivity and specificity of 73% and 39%, respectively. [19]. In a meta-analysis by Hajibandeh and colleagues, they deduced that NLR can predict both the diagnosis and severity of acute appendicitis at cut-off values of >4.7 and >8.8, respectively, thus guiding the management approach [18]. Similarly, Begic-Kapetanovic et al. suggested that the NLR could be used as a simple and reliable test in the diagnosis and prediction of complications of acute appendicitis in children [20]. Prasetya et al. exclusively studied the role of NLR in pediatric appendicitis and also concluded that NLR shows a high accuracy for diagnosis of acute appendicitis and distinguishing complicated appendicitis from simple one [21]. Likewise, in our patients, preoperative NLR was significantly higher in patients with complicated appendicitis on operative findings, thereby rendering it a sensitive marker for subclassifying this group of patients. This has two-fold implications; identification of cases suitable for NOM and relocating resources by selecting which cases to prioritize for surgery in patients with overt clinical presentation and equivocal ultrasonography. It would also be possible to predict which patients would require operative assistance from a more senior surgeon. Although our primary focus was analyzing its role in predicting postoperative complications, we believe that pediatric surgeons should familiarize themselves with NLR as a helpful tool in identifying patients who need surgery, in the setting of an unreliable history and physical examination due to communication barriers.

Several studies have identified various clinical variables as risk factors for the development of complications like surgical site infection, intra-abdominal abscesses, and prolonged postoperative ileus; these include delayed or night operations, longer duration of surgery, open appendectomy, and complicated appendicitis [22]. The incidence of complications in our patients was 21.4%; this is somewhat higher than that found in the literature possibly because complicated appendicitis affected 38.5% of our patients and three-fourths of these constituted the complication group postoperatively. Although it might make sense to predict the development of complications in these patients based on operative findings alone, it is the remaining one-fourth subset that is frequently overlooked and needs to be identified. In 2019, Delgado-Miguel et al. analyzed the usefulness of NLR as a predictor of postoperative intra-abdominal abscess in children after appendectomy and estimated an NLR cut-off point > 10.5, with a sensitivity and specificity of 85% and 75.2%, respectively [9]. Our study determined the role of NLR as a sensitive predictor of the development of complications at a cut-off value of >9.53 with a sensitivity and specificity of 79.3% and 95%, respectively, which was superior to other laboratory parameters such as leucocytosis, neutrophilia, and CRP. We also noted that the PLR predicted complications at a cut-off value >166.01 with equal sensitivity to that of NLR and a specificity of 82.1%. Interestingly, there was no significant association between the duration or type of surgery and subsequent development of complications; however, wound dehiscence was exclusively seen in the open appendectomy group.

In our institution, there is a mismatch of resources with the sheer volume of patients. It is not unusual for the number of pending emergency cases to exceed the capacity of emergency theatres. The majority of our patients come from far-off areas with limited access to health care, which explains the delayed presentations. Adherence to an antibiotic course, wound care, and timely follow-ups are frequently problematic. We diligently educate parents and guardians about postsurgical care at discharge; however, it is not rare to see them miss subtle and sometimes more obvious symptoms and signs of a complicated postoperative course. Illiteracy, low socioeconomic status, and multiparity continue to be a challenge in our society hence we prefer keeping a close watch on high-risk patients; however, considering limited resources and already overpopulated wards, we cannot afford to keep all of them in-patient. Deciding which patients can be safely discharged and which patients need close surveillance (being at high risk of developing complications) becomes important not only to prevent morbidity and mortality but also to reduce the burden of readmissions and reoperations.

NLR is a marker of systemic inflammatory response and it can be easily obtained from differential TLC count. An elevated NLR indicates both an enhanced neutrophil-mediated inflammatory reaction and a reduced lymphocyte-dependent immune response, which could influence the emergence of infectious complications after surgery. Consequently, it may be debated that patients having a higher NLR may benefit from preoperative antibiotics of a broader spectrum than those having a lower NLR. Similarly, in patients experiencing fever and diarrhea after surgery and having a higher preoperative NLR, early imaging should be considered to rule out intra-abdominal abscesses.

Limitations

Our study has certain limitations as it is a single-center study with a sample size that is relatively small, therefore the results cannot be generalized over the whole population. It is worth noting that the timing of the test can significantly alter its sensitivity and specificity as with any marker of inflammation. Results may be normal at presentation only to rise a few hours later, hence it is important to interpret these tests with a pragmatic approach. We recognize this limitation of our study, however, we believe that the clinical significance of NLR justifies further investigation with a larger multicentric sample. The strength of our study is its prospective design. All surgeries were performed by a group of surgeons having comparable skill levels with the same resources hence eliminating bias due to prolonged operation time or tissue handling. In the future, we also intend to undertake further prospective studies examining the usefulness of various antimicrobial protocols in connection to the probability of developing complications as predicted by the preoperative NLR values.

## Conclusions

Preoperative NLR can be used as a sensitive marker for distinguishing complicated from uncomplicated appendicitis in pediatric patients, which becomes useful when prioritizing cases for operative management and for relocating resources and senior expertise. Additionally, our study demonstrated that the NLR followed by PLR was the most sensitive predictor of postoperative complications after open or laparoscopic appendectomy for pediatric acute appendicitis. Although more research is needed to confirm its usefulness, observing a high NLR in children with acute appendicitis before appendectomy may help identify patients who are likely to experience complications after surgery and thus might aid in anticipating or preventing postoperative infectious complications.
